# Supplementary material for: Relaxation of mitochondrial hyperfusion in the diabetic retina via N6-furfuryladenosine confers neuroprotection regardless of glycaemic status
Source: Nat Commun. 2024 Feb 6;15:1124. doi: 10.1038/s41467-024-45387-9 (PMC10847490; doi:10.1038/s41467-024-45387-9)
Supplement: Supplementary file 1 — Supplementary Information [file 41467_2024_45387_MOESM1_ESM.pdf]

Relaxation of mitochondrial hyperfusion in the diabetic retina via N6-furfuryladenosine confers neuroprotection regardless of glycaemic status.

Aidan Anderson[1#], Nada Alfahad[1], Dulani Wimalachandra[1], Kaouthar Bouzinab[1], Paula Rudzinska[1], Heather Wood[1], Isabel Fazey[1], Heping Xu[2], Timothy J. Lyons[3,4], Nicholas M. Barnes[5], Parth Narendran[6], Janet M. Lord[1], Saaeha Rauz[1,7], Ian G. Ganley[8], Tim M. Curtis[2], Graham R. Wallace[1] and Jose R. Hombrebueno[1*#].

[1]Institute of Inflammation and Ageing, University of Birmingham, Birmingham, UK

[2]Wellcome-Wolfson Institute for Experimental Medicine, Queen's University Belfast, UK

[3]Division of Endocrinology and Diabetes, Medical University of South Carolina, Charleston, SC, USA.

[4] Diabetes Free South Carolina, Columbia, SC, USA

[5]Institute of Clinical Sciences, University of Birmingham, Birmingham, UK

[6]Institute of Immunology and Immunotherapy, University of Birmingham, Birmingham, UK

[7]Birmingham & Midland Eye Centre, Birmingham, UK

[8]MRC Protein Phosphorylation and Ubiquitylation Unit, University of Dundee, UK

*Correspondence to Dr Jose R Hombrebueno; j.m.romero@bham.ac.uk

#Aidan Anderson and Jose R. Hombrebueno contributed equally to this work.

Institute of Inflammation and Ageing, College of Medical and Dental Sciences, University of Birmingham, Edgbaston, Birmingham, B15 2TT, UK. Phone: +44(0) 1213713226

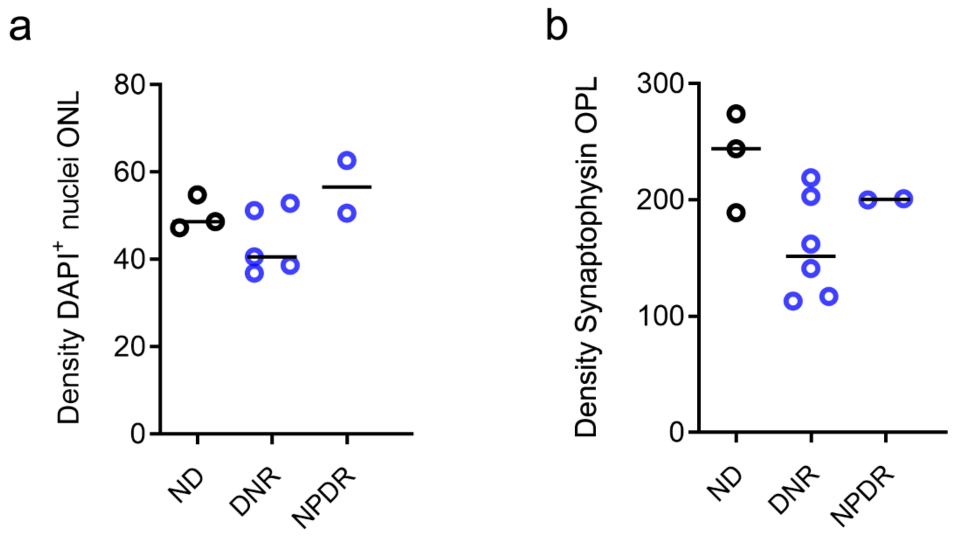

Supplemental Fig 1. Neuronal impairment in the human (outer) retina is not associated with the clinical stage of diabetic retinopathy. The density of **(a)** DAPI$^+$ nuclei at the outer nuclear layer (ONL) and **(b)** synaptophysin$^+$ processes at the outer plexiform layer (OPL) in human retinas from non-diabetic (ND; n=3 donor eyes), diabetic with no retinopathy (DNR; n=5 donor eyes [a], n=6 donor eyes [b]), and non-proliferative diabetic retinopathy (NPDR; n=2 donor eyes). Data are presented as dot-plots with median values for each group.

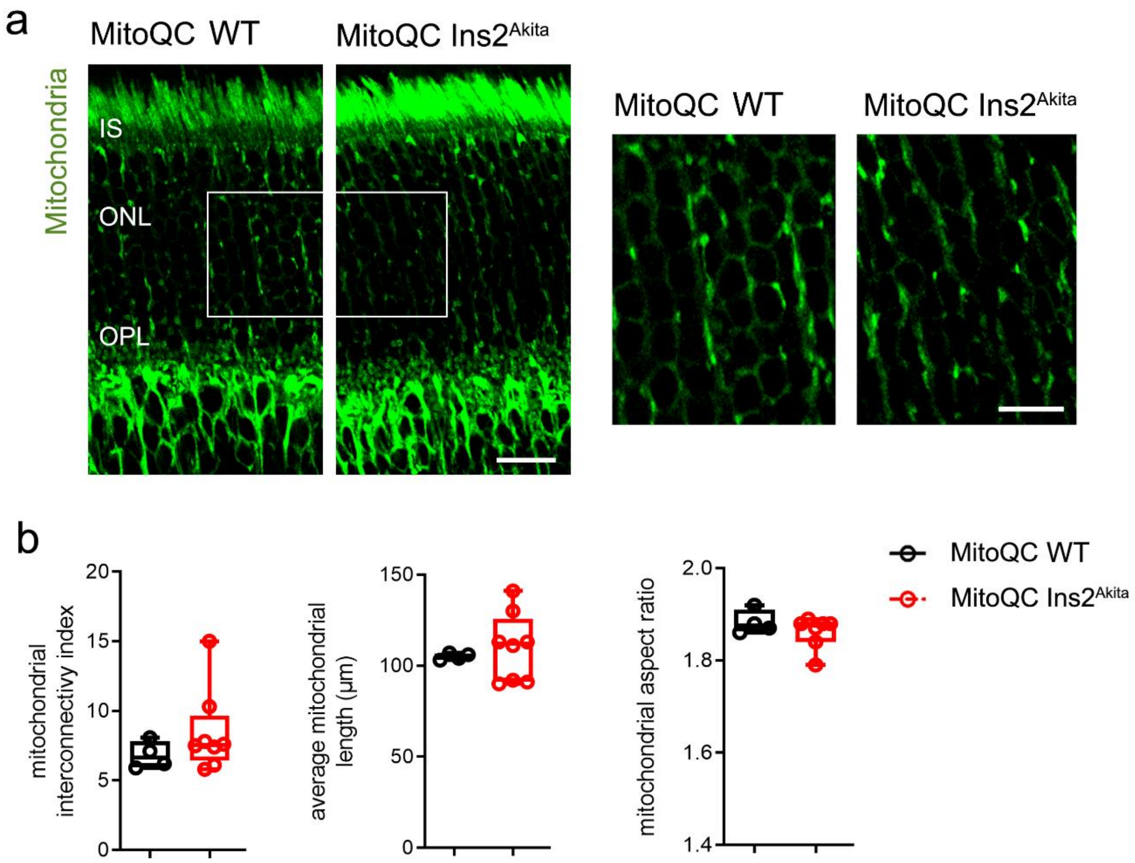

Supplemental Fig 2. Mitochondrial morphology is not altered in the Ins2^{Akita} outer retina by 3-months of diabetes. (a-b) Morphometric quantification of mitochondrial fusion (Fis1-GFP signal) in the ONL of 3-month diabetic mitophagy reporter mice (mitoQC Ins2^{Akita} males) and age-matched non-diabetic male siblings (mitoQC WT). The inset rectangle shows a high-magnification view of mitochondrial morphology at the ONL. **(b)** Mitochondrial interconnectivity index, average mitochondrial length and mitochondrial aspect ratio (AR). Eyes per strain and condition: mitoQC WT (n=4), mitoQC Ins2^{Akita} (n=8 [interconnectivity index, average mitochondrial length], n=7 [AR]). Data are presented in box-and-whisker plots with single data points (for definition of boxplot elements see “Methods” section). IS, photoreceptor inner segments; ONL, the outer nuclear layer; OPL, outer plexiform layer. Scale bars: 40 µm (**a**), 20 µm (inset).

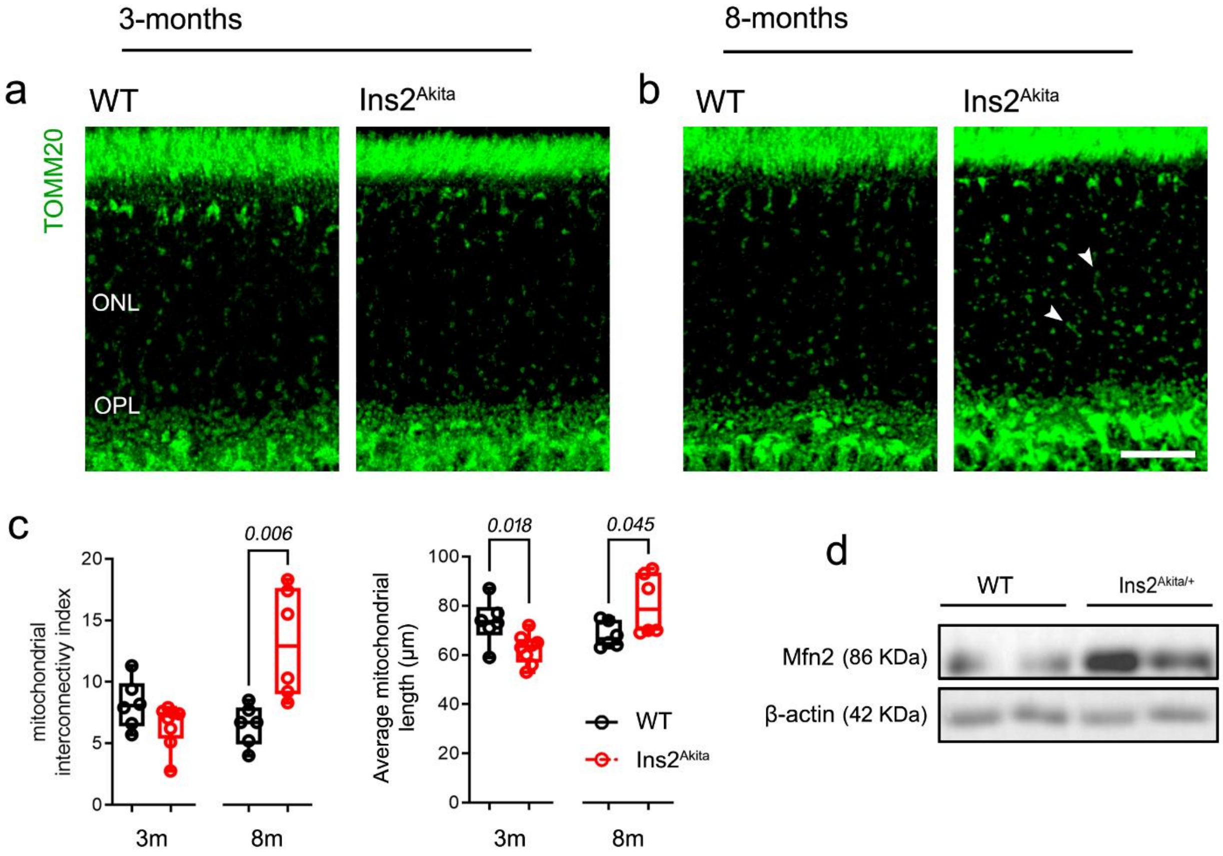

Supplemental Fig 3. Mitochondria remodels towards hyperfusion in the Ins2^{Akita} outer retina during diabetes progression. (a-c) Morphometric quantification of mitochondrial fusion (TOMM20 immunostaining) in the ONL of 3-month and 8-month diabetic Ins2^{Akita} male mice and age-matched WT male siblings. **(c)** Mitochondrial interconnectivity index and average mitochondrial length in different treatment groups. Eyes per strain and age: mitoQC WT 3m (n=6), mitoQC WT 8m (n=6), mitoQC Ins2^{Akita} 3m (n=8), mitoQC Ins2^{Akita} 8m (n=6). **(d)** Example immunoblot of Mitofusin-2 (Mfn2) and β-actin loading controls in retinal lysates of 8-month diabetic Ins2^{Akita} male mice and age-matched WT male siblings (n=2 eyes/strain). Data are presented in box-and-whisker plots with single data points (for definition of boxplot elements see "Methods" section). *P-values* were calculated using 2-sided unpaired Student's t-test between mice of same strain and age. ONL, outer nuclear layer; OPL, outer plexiform layer. Scale bar: 40 µm.

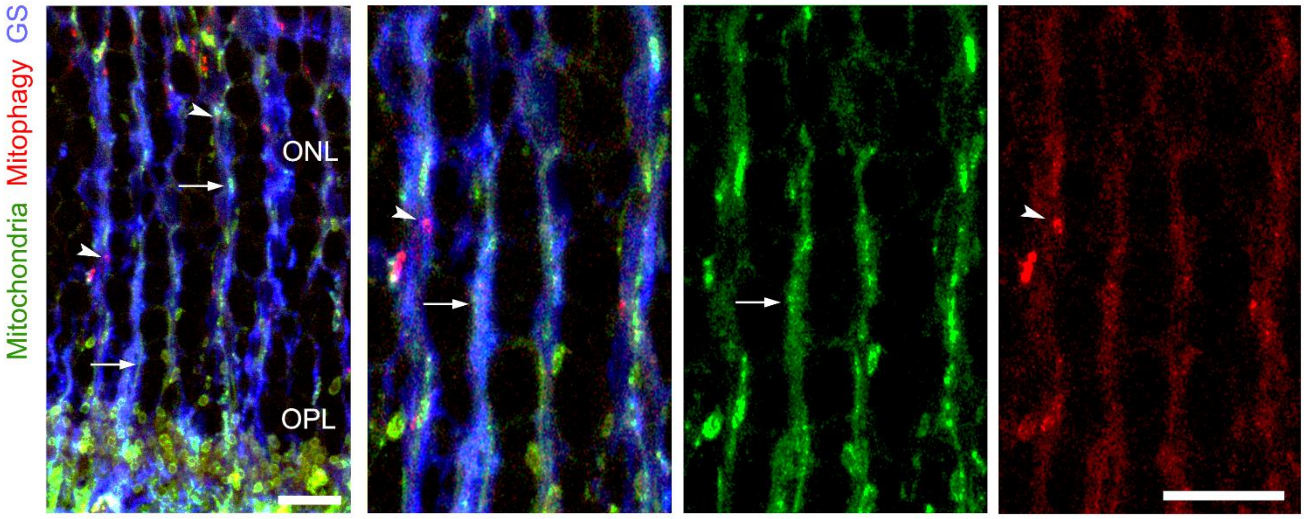

Supplemental Fig 4. Retinal Müller glia are involved in the regulation of mitochondrial turnover at the outer retina. Retinal micrographs of 3-month mitophagy reporter mice (mitoQC) mice processed for glutamine synthase (GS) immunostaining. A substantial fraction of filamentous mitochondria (arrows) and mitolysosomes (arrowheads) are found within GS^+ Müller glia processes at the outer nuclear layer (ONL). OPL, outer plexiform layer. Scale bar: 20 µm.

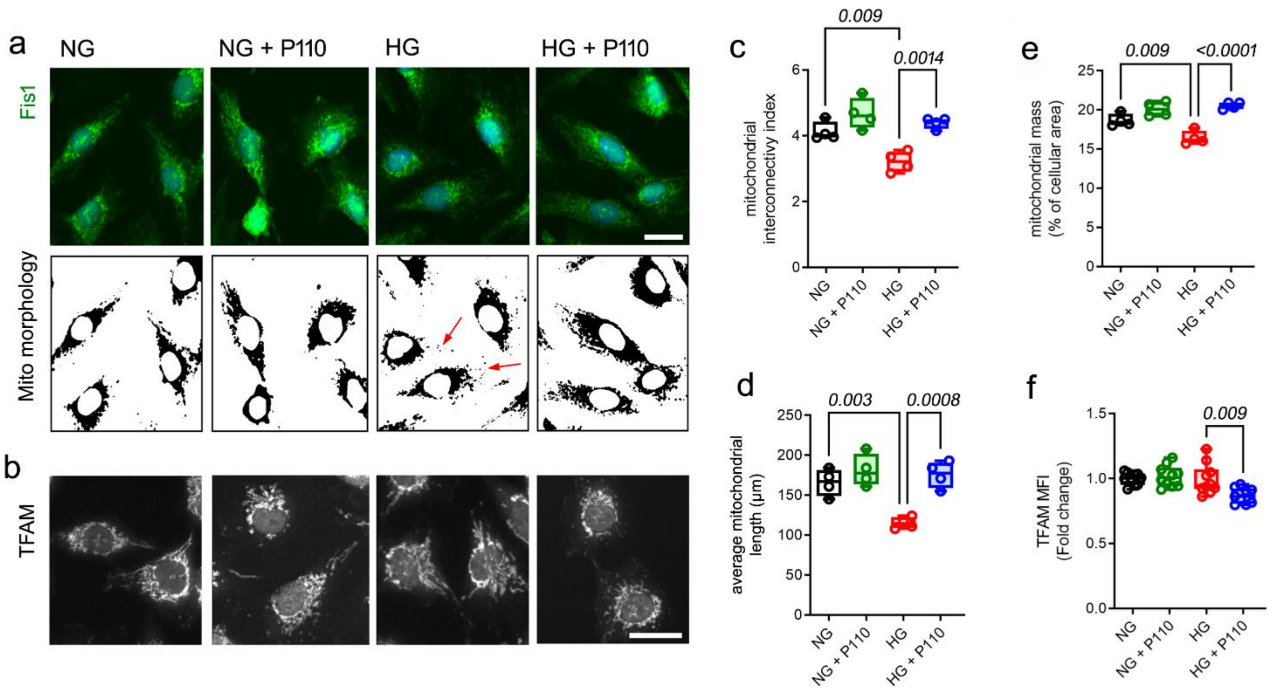

Supplemental Fig 5. Exacerbated fusion impairs biogenesis machinery in human MIO-M1 Müller glia under hyperglycaemia. Human MIO-M1 Müller cells were antagonized for mitochondrial fission using a Drp1-inhibitor peptide (P110) under physiological (NG; 5.5mM) or elevated glucose (HG; 30.5mM) conditions (see also Fig 3a), and then evaluated for mitochondrial morphology (Fis1 immunostaining) or mitochondrial biogenesis adaptors (TFAM). **(a-b)** Representative micrographs of MIO-M1 Müller cultures immunostained and binarized for Fis1 or TFAM in different treatment groups. Arrows indicate mitochondrial fragmentation. **(c-d)** Morphometric analysis of mitochondrial fusion (interconnectivity and average mitochondrial length). **(e)** Mitochondrial mass (% of Fis1^{+} signal per cell). **(f)** TFAM expression (mean fluorescence intensity [MFI]). NG, NG + P110, HG (n=4 [c-e], n =10 [f]), HG + P110 (n=4 [c-e], n =9 [f]) independent replicates. Data are presented in box-and-whisker plots with single data points (for definition of boxplot elements see "Methods" section). *P-values* were calculated using One-way ANOVA with Dunnett's multiple comparison. TFAM, mitochondrial transcription factor A. Scale bars: 20 µm.

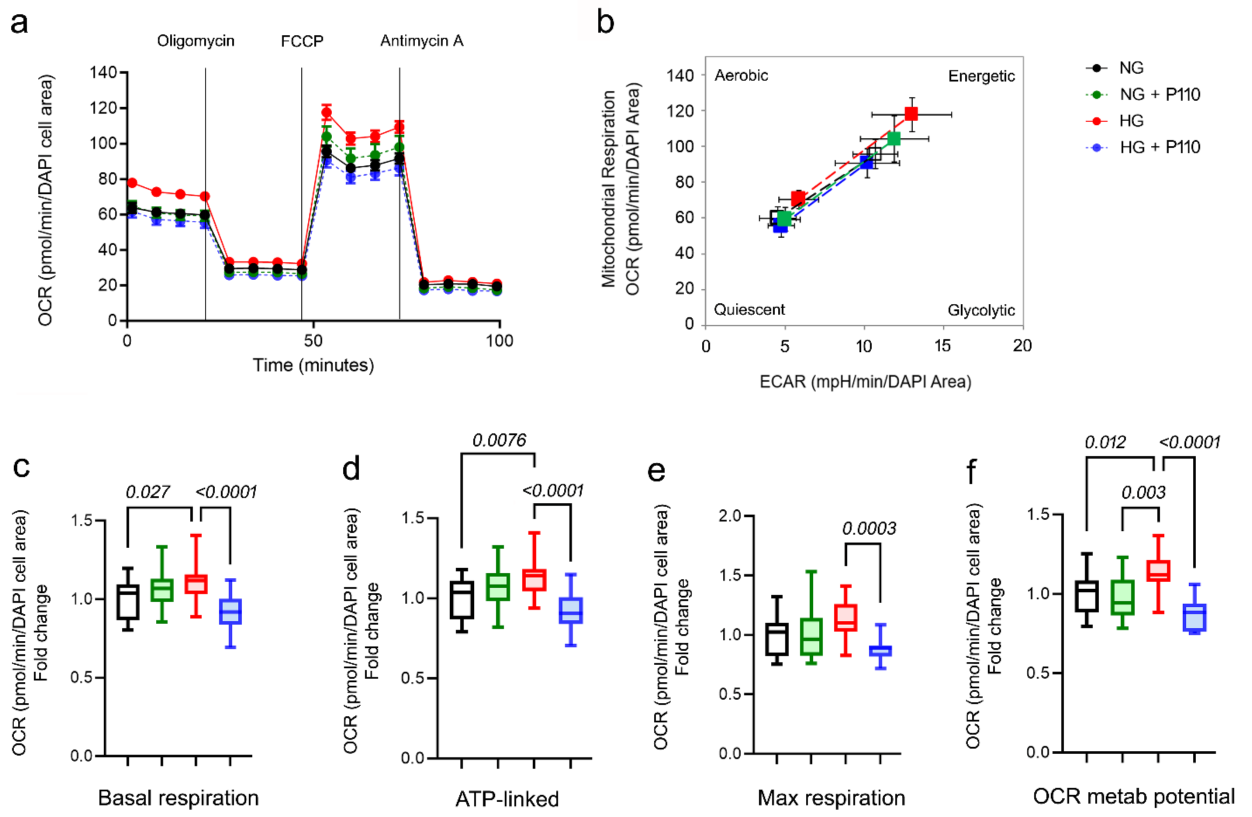

Supplemental Fig 6. Exacerbated fusion impairs mitochondrial bioenergetics in mouse primary Müller glia under hyperglycaemia. Mouse primary Müller cells were antagonized for mitochondrial fission using a Drp1-inhibitor peptide (P110) under physiological (NG; 5.5mM) or elevated glucose (HG; 30.5mM) conditions (see also Fig 3a), and then evaluated for mitochondrial bioenergetics using Seahorse. Representative Seahorse assay of **(a)** metabolic flux and **(b)** metabolic potential using *Cell Mito Stress Test*. **(c-f)** Quantification of oxygen consumption rate (OCR) indicative of **(c)** basal respiration, **(d)** ATP-linked respiration, **(e)** Maximum respiration and **(f)** metabolic potential. NG, NG + P110, HG (n=17), HG + P110 (n=15) independent replicates. **(c-f)**. Data are presented in **(a-b)** mean ± SE or **(c-f)** box-and-whisker plots (for definition of boxplot elements see “Methods” section). *P-values* were calculated using One-way ANOVA with Dunnett’s multiple comparison. ECAR, extracellular acidification rate.

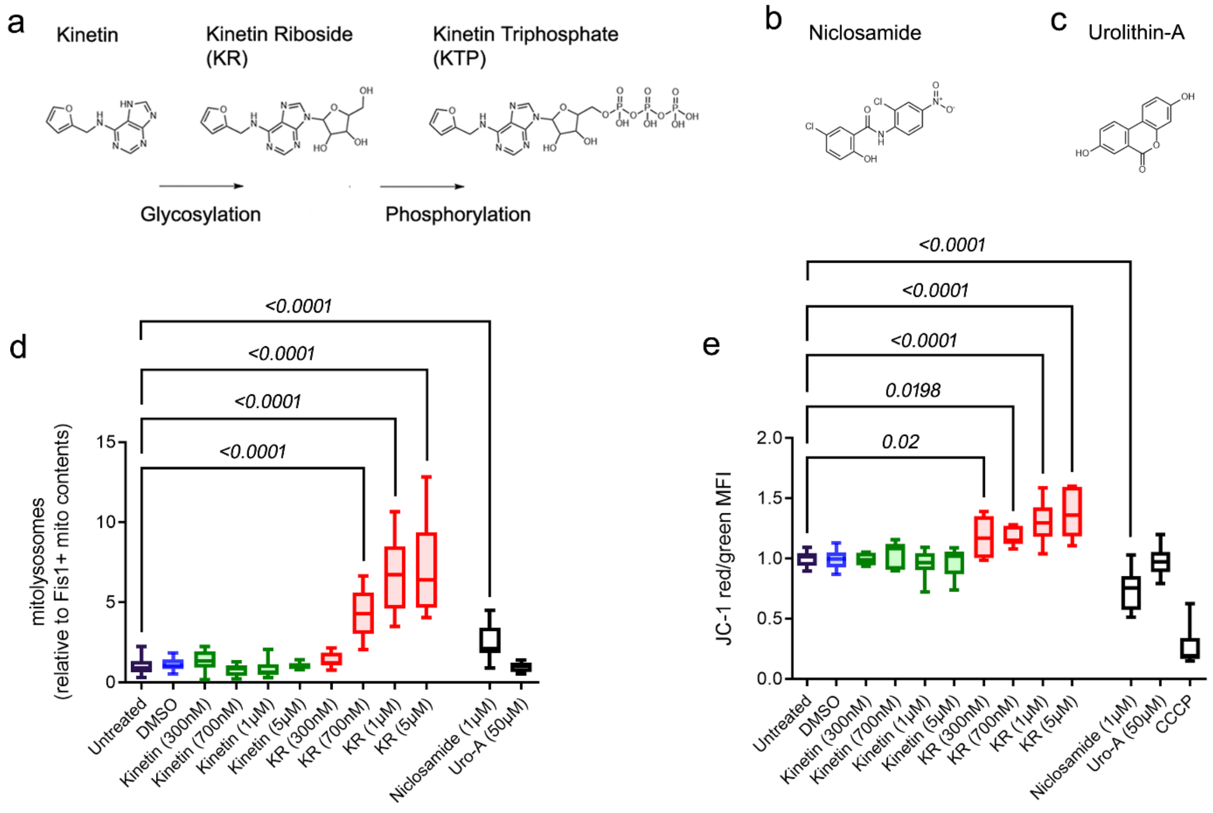

Supplemental Fig 7. Glycosylated N6-furfuryladenine (kinetin riboside) but not its precursor (kinetin) amplifies mitophagy in Müller glia independent of mitochondrial uncoupling. Primary Müller cells isolated from mitoQC mice (MitoQC PMCs) or human MIO-M1 cells were treated with putative activators of PINK1 mitophagy including **(a)** kinetin and its glycosylated metabolite kinetin riboside (KR) or **(b)** niclosamide or **(c)** urolithin-A (Uro-A). **(d)** Quantification of mitophagy in MitoQC PMCs after 24h of treatment. n=7 (KR 300nM), n=8 (Kinetin 300nM, Kinetin 700nM, Kinetin 5µM, KR 700nM, KR 5µM, niclosamide 1µM, Uro-A 50µM), n=16 (Kinetin 1µM, KR 1µM), n =18 (untreated), n=19 (DMSO) independent replicates. **(e)** Evaluation of mitochondrial membrane potential (ψm) by JC-1 dye (red, hyperpolarized; green, depolarized mitochondria) in human MIO-M1 cells 24h after treatments. CCCP (100 µM) was added as a mitochondrial uncoupler positive control (2 hours). n=6 (Kinetin 300nM, Kinetin 700nM, Kinetin 5µM, KR 300nM, KR 700nM, KR 5µM), n=7 (Uro-A 50µM), n=8 (niclosamide 1µM), n=11 (Kinetin 1µM, KR 1µM), n=13 (CCCP), n=16 (untreated), n=17 (DMSO) independent replicates. Data are presented in box-and-whisker plots (for definition of boxplot elements see "Methods" section). *P-values* were calculated using One-way ANOVA with Dunnett's multiple comparisons.

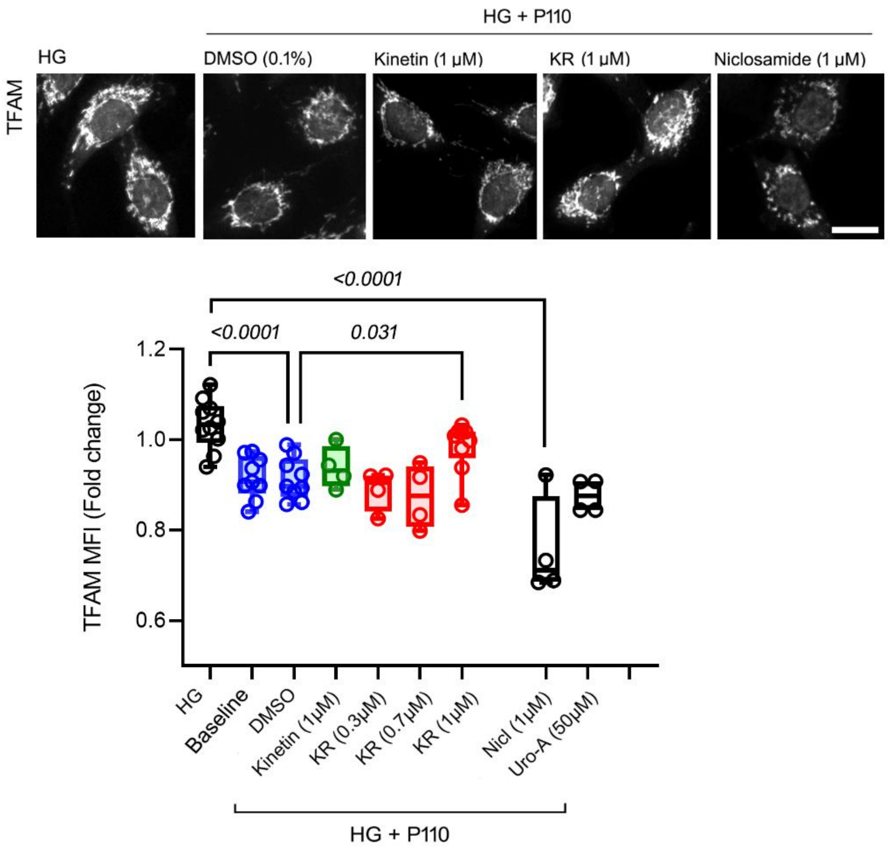

Supplemental Fig 8. Kinetin riboside but not its precursor (kinetin) rescues mitochondrial biogenesis machinery in Müller glia under diabetes-mitochondrial hyperfusion. Human MIO-M1 cells were antagonized for mitochondrial fission (P110 peptide) under elevated glucose (HG; 30.5mM) conditions (see also Fig 3a), and treated with different concentrations of kinetin, kinetin riboside (KR), niclosamide (Nicl), urolithin-A (Uro-A) or DMSO control (0.1%). Following treatment, the expression levels of mitochondrial biogenesis adaptor TFAM was evaluated by immunocytochemistry. n=4 (Kinetin 1µM, KR 0.3µM, KR 0.7µM, Nicl 1µM, Uro-A 50µM), n=9 (Baseline, DMSO, KR 1µM), n=10 (HG) independent replicates. Data are presented in box-and-whisker plots with single data points (for definition of boxplot elements see "Methods" section). *P-values* were calculated using One-way ANOVA with Dunnett's multiple comparisons. TFAM, mitochondrial transcription factor A; MFI, mean fluorescence intensity. Scale bar: 20 µm.

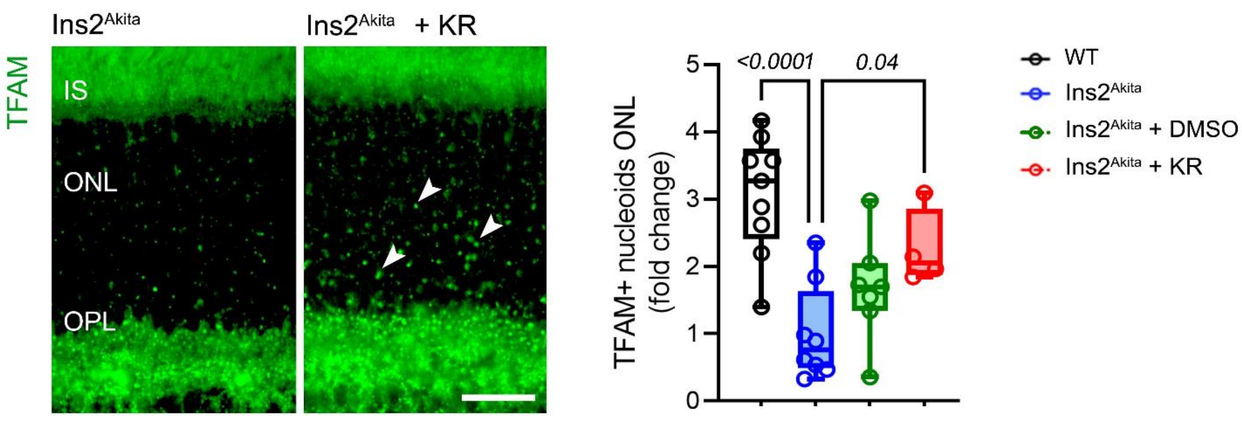

Supplemental Fig 9. Kinetin riboside (KR) rescues mitochondrial biogenesis nucleoids in diabetic Ins2^{Akita} retinas. KR (60 mg/L) or DMSO vehicle-control (0.1%) was supplemented in the drinking water from 4-months to 8-months of diabetes in Ins2^{Akita} male mice. Following treatment, the density of TFAM^{+} mitochondrial nucleoids at the outer retina (arrowheads) was evaluated via immunohistochemistry and compared between diabetic and non-diabetic (WT) age-matched male groups. Eyes per strain and condition: WT (n=9), Ins2^{Akita} (n=8), Ins2^{Akita} + DMSO (n=7), Ins2^{Akita} + KR (n=4). Data are presented in box-and-whisker plots with single data points (for definition of boxplot elements see "Methods" section). *P-values* were calculated using One-way ANOVA with Dunnett's multiple comparison. IS, photoreceptor inner segments, ONL, outer nuclear layer; OPL, outer plexiform layer, TFAM, mitochondrial transcription factor A. Scale bar: 40 μm.

Supplemental Table 1. Primary antibodies used for western blot (WB), immunohistochemistry (IHC) and immunocytochemistry (IC).

**Antigen**	**Antiserum (Host)**	**Dilution**	**Source (catalogue number)**
ATP Synthase (beta)	mouse	1:1000 (WB)	Thermo (A-21351)
β-actin	mouse	1:10000 (WB)	Santa Cruz (sc-47778)
β-III tubulin	rabbit	1:500 (IC)	Abcam (ab18207)
Calbindin	rabbit	1:1000 (IHC)	Swant (CB-38a)
Cone-arrestin	rabbit	1:10000 (IHC)	Chemicon (ab15282)
Cox4	goat	1:50 (IHC)	R&D systems (AF5814)
Fis1	rabbit	1:500 (IHC and IC)	Genetex (GTX111010)
GABA	rabbit	1:500 (IHC)	Sigma (A2052)
Glutamine synthase	rabbit	1:10000 (IHC)<br>1:500 (IC)	Sigma (G2781)
Neurofilament (heavy chain)	rabbit	1:500 (IC)	Proteintech (21471-1-AP)
Mitofusin-2	rabbit	1:1000 (WB)	Cell Signaling (9482S)
M-opsin	rabbit	1:1000 (IHC)	Chemicon (AB5405)
Synaptophysin	rabbit	1:500 (IHC)	Abcam (ab14692)
TFAM	rabbit	1:500 (IHC and IC)	Genetex (GTX112760)
TOMM20	rabbit	1:500 (IHC)	Sigma (HPA011562)
Vimentin	rabbit	1:500 (IHC)	Abcam (ab92547)

Supplemental Table 2. Secondary antibodies used for western blot (WB), immunohistochemistry (IHC) and immunocytochemistry (IC).

**Type**	**Antiserum (Host)**	**Dilution**	**Source (catalogue number)**
Anti-Rabbit IgG Alexa Fluor™ Plus 405	Donkey	1:400 (IHC and IC)	Thermo (A48258)
Anti-Rabbit IgG Alexa Fluor™ Plus 488	Donkey	1:400 (IHC and IC)	Thermo (A32790)
Anti-Rabbit IgG Alexa Fluor™ Plus 594	Donkey	1:400 (IHC and IC)	Thermo (A32754)
Anti-Goat Alexa Fluor® 488 AffiniPure™ IgG	Donkey	1:400 (IHC and IC)	Jackson ImmunoResearch (705-545-147)
Anti-Mouse IgG Secondary Antibody, HRP	Goat	1:500 (WB)	Thermo (32430)
Anti-Rabbit IgG Secondary Antibody, HRP	Goat	1:4000 (WB)	Thermo (656120)
