# Supplementary material for: Relaxation of mitochondrial hyperfusion in the diabetic retina via N6-furfuryladenosine confers neuroprotection regardless of glycaemic status
Source: Nat Commun. 2024 Feb 6;15:1124. doi: 10.1038/s41467-024-45387-9 (PMC10847490; doi:10.1038/s41467-024-45387-9)
Supplement: Supplementary file 3 — Reporting Summary [file 41467_2024_45387_MOESM3_ESM.pdf]

nature portfolio

Corresponding author(s): Jose R Hombrebueno
Last updated by author(s): Jan 16, 2024

Reporting Summary

Nature Portfolio wishes to improve the reproducibility of the work that we publish. This form provides structure for consistency and transparency in reporting. For further information on Nature Portfolio policies, see our Editorial Policies and the Editorial Policy Checklist.

Statistics

For all statistical analyses, confirm that the following items are present in the figure legend, table legend, main text, or Methods section.

n/a	Confirmed	
☐	☒	The exact sample size (n) for each experimental group/condition, given as a discrete number and unit of measurement
☐	☒	A statement on whether measurements were taken from distinct samples or whether the same sample was measured repeatedly
☐	☒	The statistical test(s) used AND whether they are one- or two-sided<br>*Only common tests should be described solely by name; describe more complex techniques in the Methods section.*
☒	☐	A description of all covariates tested
☐	☒	A description of any assumptions or corrections, such as tests of normality and adjustment for multiple comparisons
☐	☒	A full description of the statistical parameters including central tendency (e.g. means) or other basic estimates (e.g. regression coefficient) AND variation (e.g. standard deviation) or associated estimates of uncertainty (e.g. confidence intervals)
☐	☒	For null hypothesis testing, the test statistic (e.g. F, t, r) with confidence intervals, effect sizes, degrees of freedom and P value noted<br>*Give P values as exact values whenever suitable.*
☒	☐	For Bayesian analysis, information on the choice of priors and Markov chain Monte Carlo settings
☒	☐	For hierarchical and complex designs, identification of the appropriate level for tests and full reporting of outcomes
☒	☐	Estimates of effect sizes (e.g. Cohen's d, Pearson's r), indicating how they were calculated

Our web collection on statistics for biologists contains articles on many of the points above.

Software and code

Policy information about availability of computer code

Data collection	Images at confocal and epifluorescence microscopy were acquired using Nikon EZ-C1 Software and Image-Pro Plus respectively.<br><br>xPONENT software was used for Luminex data collection.<br><br>Syngene’s GeneSys software was used for immunoblot data collection.<br><br>Espion Visual Electrophysiology System was used to acquire scotopic electroretinogram recordings.<br><br>The Spectralis-Heidelberg OCT system was used to obtain retinal mouse scans.<br><br>Extracellular metabolic flux data (OCR and ECAR) were acquired using the Agilent Seahorse Wave Desktop software.
Data analysis	Miicroscopy images and immunoblots densitometry were analyzed using FIJI/ImageJ software (v1.54d - a detailed description for each analysis is shown in methods). The AngioTool plugin (v 0.5a) was used to evaluate the morphology of the mitochondrial network.<br><br>The Spectralis-Heidelberg OCT system was used to analyze retinal mouse scans.<br><br>Extracellular metabolic flux data was evaluated using Seahorse Wave Desktop Software

Graph Prism software (v10.1.0) was used to perform statistical analysis and generate graphs.

For manuscripts utilizing custom algorithms or software that are central to the research but not yet described in published literature, software must be made available to editors and reviewers. We strongly encourage code deposition in a community repository (e.g. GitHub). See the Nature Portfolio guidelines for submitting code & software for further information.

Data

Policy information about availability of data

All manuscripts must include a data availability statement. This statement should provide the following information, where applicable:

- Accession codes, unique identifiers, or web links for publicly available datasets
- A description of any restrictions on data availability
- For clinical datasets or third party data, please ensure that the statement adheres to our policy

All source data are provided with this paper.

Research involving human participants, their data, or biological material

Policy information about studies with human participants or human data. See also policy information about sex, gender (identity/presentation), and sexual orientation and race, ethnicity and racism.

Reporting on sex and gender	Male and female, diabetic and non-diabetic donors were included in the study - however we did not select on the basis of sex or gender for human tissue analysis given the limited number of samples in the non-diabetic (n=3, all males between 70-72 years old) and the diabetic group (n=8, 2 females, 4 males, 2 unknown between 59-80 years old)
Reporting on race, ethnicity, or other socially relevant groupings	We did not consider race, ethnicity, or other socially relevant groupings for human tissue analysis.
Population characteristics	National Disease Research Interchange (NDRI) eye tissues were obtained from Diabetic individuals ranging from no retinopathy (n=6) to mild non-proliferative retinopathy (n=2). Non-diabetic donors had no known history of ocular disease. All donors (diabetic and non-diabetic) were age-matched between 59-80 year-old (see above).
Recruitment	NDRI eye tissues were obtained from Diabetic individuals ranging from no retinopathy to mild non-proliferative retinopathy. Individuals who had had any treatment for diabetic retinopathy (e.g., laser photocoagulation or intra-ocular anti-angiogenic injections) were excluded from the study.
Ethics oversight	Human studies were approved by Ethics Committee of Oklahoma Health Sciences Centre (OUHSC) and conducted according to the Declaration of Helsinki principles. All donors provided written informed consent.

Note that full information on the approval of the study protocol must also be provided in the manuscript.

Field-specific reporting

Please select the one below that is the best fit for your research. If you are not sure, read the appropriate sections before making your selection.

☒ Life sciences ☐ Behavioural & social sciences ☐ Ecological, evolutionary & environmental sciences

For a reference copy of the document with all sections, see nature.com/documents/nr-reporting-summary-flat.pdf

Life sciences study design

All studies must disclose on these points even when the disclosure is negative.

Sample size	For in vivo experiments, sample size was adjusted to n = 4–10 eyes in each mouse group based on an 80% power and a 5% significant level to compare the difference between 2-4 means. For in vitro experiments we did not use any criteria to determine the sample size. As much data as possible was collected depending on the nature of the experiments or in order to obtain meaningful statistical analysis (at least N ≥ 3 independent replicates were used).
Data exclusions	No data exclusions in this study.
Replication	For human and animal studies, at least 3 independent biological replicates were performed (unless differently stated in the fig legends). For cell studies at least N ≥ 3 independent replicates were used. All attempts for replication were successful.
Randomization	Age-matched mouse males were randomly assigned into each experimental group. For In vitro studies, cells or conditions were assigned randomly to each experimental group.
Blinding	Investigators were not always blinded to group allocation during data collection and analysis because no subjective evaluations were required. However, the acquisition and quantification of images were standardized to eliminate any bias from the analysis. The regions of the retina selected for confocal imaging were based on the DAPI nuclear signal and not the marker of interest (e.g., mitolysosomes, Cone arrestin, GABA, etc). Most image analysis was performed automatically using FIJI software, including measurements of mean fluorescence intensities (MFIs), mitochondrial morphology and particle analysis. Images from mouse retinas were invariably obtained from the same retinal eccentricity (mid-

centre) and then averaged in each eye for statistical analysis. For in vitro cell culture work, regions of interest were selected using brightfield imaging or nuclear DAPI (without checking the fluorescent signal corresponding to the marker of interest prior to acquisition) in 4 cardinal points of the well.

Reporting for specific materials, systems and methods

We require information from authors about some types of materials, experimental systems and methods used in many studies. Here, indicate whether each material, system or method listed is relevant to your study. If you are not sure if a list item applies to your research, read the appropriate section before selecting a response.

Materials & experimental systems

n/a	Involved in the study
☐	☒ Antibodies
☐	☒ Eukaryotic cell lines
☒	☐ Palaeontology and archaeology
☐	☒ Animals and other organisms
☒	☐ Clinical data
☒	☐ Dual use research of concern
☒	☐ Plants

Methods

n/a	Involved in the study
☒	☐ ChIP-seq
☒	☐ Flow cytometry
☒	☐ MRI-based neuroimaging

Antibodies

Antibodies used

Primary Abs:

ATP Synthase (beta) - Thermo (A-21351): 1:1000 (immunoblot)
β-actin - Santa Cruz (sc-47778): 1:10000 (immunoblot)
β-III tubulin- Abcam (ab18207): 1:500 (immunocytochemistry)
Calbindin - Swant (CB-38a): 1:1000 (immunohistochemistry)
Cone-arrestin - Chemicon (ab15282): 1:10000 (immunohistochemistry)
Cox4 - R&D systems (AF5814): 1:50 (immunohistochemistry)
Fis1 - Genetex (GTX111010): 1:500 (immunohisto/cytochemistry)
GABA - Sigma (A2052): 1:500 (immunohistochemistry)
Glutamine synthase - Sigma (G2781): 1:10000 (immunohistochemistry); 1:500 (immunocytochemistry)
Neurofilament (heavy chain) - Proteintech (21471-1-AP): 1:500 (immunocytochemistry)
Mitofusin-2 - Cell Signaling (9482S): 1:1000 (immunoblot)
M-opsin - Chemicon (AB5405): 1:1000 (immunohistochemistry)
Synaptophysin - Abcam (ab14692): 1:500 (immunohistochemistry)
TFAM - Genetex (GTX112760): 1:500 (immunohisto/cytochemistry)
TOMM20 - Sigma (HPA011562): 1:500 (immunohistochemistry)
Vimentin - Abcam (ab92547): 1:500 (immunohistochemistry)

Secondary Abs:

Donkey anti-Rabbit IgG (H+L) Alexa Fluor™ Plus 405 - Thermo (A48258): 1:400 (immunohisto/cytochemistry)
Donkey anti-Rabbit IgG (H+L) Alexa Fluor™ Plus 594 - Thermo (A32754): 1:400 (immunohisto/cytochemistry)
Donkey anti-Rabbit IgG (H+L) Alexa Fluor™ Plus 488 - Thermo (A32790): 1:400 (immunohisto/cytochemistry)
Donkey Anti-Goat Alexa Fluor® 488 AffiniPure™ IgG - Jackson ImmunoResearch (705-545-147): 1:400 (immunohisto/cytochemistry)
Goat anti-Mouse IgG (H+L) Secondary Antibody, HRP - Thermo (32430): 1:500 (immunoblot)
Goat anti-Rabbit IgG (H+L) Secondary Antibody, HRP - Thermo (656120): 1:4000 (immunoblot)

Validation

All the antibodies are commercially available and validated by manufacturers. The indicated species and application can be accessed on the manufacturers' website. These antibodies validation have been confirmed on human and/or mice according to the product introductions. The specific products links were as following:

ATP Synthase (beta) - https://www.thermofisher.com/antibody/product/ATP-Synthase-beta-Antibody-clone-3D5AB1-Monoclonal/A-21351
β-actin - https://datasheets.scbt.com/sc-47778.pdf
β-III tubulin - https://www.abcam.com/products/primary-antibodies/beta-iii-tubulin-antibody-neuronal-marker-ab18207.html
Calbindin - https://www.swant.com/pdfs/Rabbit_anti_calbindin_D-28k_CB38.pdf
Cone-arrestin - https://www.merckmillipore.com/IE/en/product/Anti-Cone-Arrestin-Antibody,MM_NF-AB15282?ReferrerURL=https%3A%2F%2Fwww.google.com%2F
Cox4 - https://www.rndsystems.com/products/human-mouse-cox4-i1-antibody_af5814
Fis1 - https://www.genetex.com/Product/Detail/FIS1-antibody/GTX111010
GABA - https://www.sigmaaldrich.com/GB/en/product/sigma/a2052
Glutamine synthase - https://www.sigmaaldrich.com/deepweb/assets/sigmaaldrich/product/documents/175/071/g2781dat.pdf

Neurofilament - https://www.ptglab.com/products/NEFH-Antibody-21471-1-AP.htm
Mitofusin-2 - https://www.cellsignal.com/products/primary-antibodies/mitofusin-2-d2d10-rabbit-mab/9482
M-opsin - https://www.merckmillipore.com/IE/en/product/Anti-Opsin-Antibody-Red-Green,MM_NF-AB5405?ReferrerURL=https%3A%2F%2Fwww.google.com%2F
Synaptophysin - https://www.abcam.com/products/primary-antibodies/synaptophysin-antibody-ab14692.html
TFAM - https://www.genetex.com/Product/Detail/mtTFA-antibody/GTX112760
TOMM20 - https://www.sigmaaldrich.com/GB/en/search/hpa011562?focus=products&page=1&perpage=30&sort=relevance&term=hpa011562&type=product
Vimentin - https://www.abcam.com/products/primary-antibodies/vimentin-antibody-epr3776-cytoskeleton-marker-ab92547.html
Donkey anti-Rabbit IgG (H+L) Alexa Fluor™ Plus 405 - https://www.thermofisher.com/antibody/product/Donkey-anti-Rabbit-IgG-H-L-Highly-Cross-Adsorbed-Secondary-Antibody-Polyclonal/A48258
Donkey anti-Rabbit IgG (H+L) Alexa Fluor™ Plus 594 - https://www.thermofisher.com/antibody/product/Donkey-anti-Rabbit-IgG-H-L-Highly-Cross-Adsorbed-Secondary-Antibody-Polyclonal/A32754
Donkey anti-Rabbit IgG (H+L) Alexa Fluor™ Plus 488 - https://www.thermofisher.com/antibody/product/Donkey-anti-Rabbit-IgG-H-L-Highly-Cross-Adsorbed-Secondary-Antibody-Polyclonal/A32790
Donkey Anti-Goat Alexa Fluor® 488 AffiniPure™ IgG - https://www.jacksonimmuno.com/catalog/products/705-545-147
Goat anti-Mouse IgG (H+L) Secondary Antibody, HRP - https://www.thermofisher.com/antibody/product/Goat-anti-Mouse-IgG-H-L-Secondary-Antibody-Polyclonal/32430
Goat anti-Rabbit IgG (H+L) Secondary Antibody, HRP - https://www.thermofisher.com/antibody/product/Goat-anti-Rabbit-IgG-H-L-Secondary-Antibody-Polyclonal/65-6120

Eukaryotic cell lines

Policy information about cell lines and Sex and Gender in Research

Cell line source(s)	MIO-M1 were kindly provided by Professor G Astrid Limb
Authentication	MIO-M1 Cell line was authenticated via expression of common Muller glia markers (Glutamine synthase, GFAP, Vimentin) (PMID: 11867609) and neural stem-cell characteristics (Fig 4G-H) (PMID: 17525239)
Mycoplasma contamination	All cell lines were negative for mycoplasma.
Commonly misidentified lines (See ICLAC register)	No commonly misidentified cell lines were used.

Animals and other research organisms

Policy information about studies involving animals; ARRIVE guidelines recommended for reporting animal research, and Sex and Gender in Research

Laboratory animals	Mouse strains (all on C57BL/6J background): Heterozygous Ins2Akita (Jackson Labs stock number 003548) and age-matched non-diabetic siblings; Mito-QC mice (mCherry-GFP-mtFIS1101–153) was kindly provided by Prof Ian G Ganley (PMID: 27458135)<br><br>All experimental mouse strains were used at 12-weeks or 36-weeks of age.
Wild animals	This study did not involve wild animals
Reporting on sex	Only males were used in this study, as the diabetic phenotype is incomplete in Heterozygous Ins2Akita females
Field-collected samples	This study did not involve samples collected from the field
Ethics oversight	The study was approved by the Ethics Committee of the University of Birmingham (Project Licence Number PP6860623) and Queen's University Belfast (Project Licence Number PPL2814. All animal procedures were approved by Ethical Review Body (AWERB) and authorized under the UK Animals (Scientific Procedures) Act 1986.

Note that full information on the approval of the study protocol must also be provided in the manuscript.

Plants

Seed stocks	N/A
Novel plant genotypes	N/A
Authentication	N/A
